# *Cis*-Regulatory Variants Affect *CHRNA5* mRNA Expression in Populations of African and European Ancestry

**DOI:** 10.1371/journal.pone.0080204

**Published:** 2013-11-26

**Authors:** Jen-Chyong Wang, Noah Spiegel, Sarah Bertelsen, Nhung Le, Nicholas McKenna, John P. Budde, Oscar Harari, Manav Kapoor, Andrew Brooks, Dana Hancock, Jay Tischfield, Tatiana Foroud, Laura J. Bierut, Joe Henry Steinbach, Howard J. Edenberg, Bryan J. Traynor, Alison M. Goate

**Affiliations:** 1 Department of Psychiatry, Washington University School of Medicine, Saint Louis, Missouri, United States of America; 2 Department of Genetics, Rutgers University, Piscataway, New Jersey, United States of America; 3 Department of Behavioral Health Epidemiology, Research Triangle Institute, Research Triangle Park, North Carolina, United States of America; 4 Department of Medical and Molecular Genetics, Indiana University School of Medicine, Indianapolis, Indiana, United States of America; 5 Department of Anesthesiology, Washington University School of Medicine, St. Louis, Missouri, United States of America; 6 Department of Biochemistry and Molecular Biology, Indiana University School of Medicine, Indianapolis, Indiana, United States of America; 7 Neuromuscular Diseases Research Unit, Laboratory of Neurogenetics, NIA, NIH, Bethesda, Maryland, United States of America; 8 Department of Genetics and Department of Neurology, Washington University School of Medicine, Saint Louis, Missouri, United States of America; MOE Key Laboratory of Environment and Health, School of Public Health, Tongji Medical College, Huazhong University of Science and Technology, China

## Abstract

Variants within the gene cluster encoding α3, α5, and β4 nicotinic receptor subunits are major risk factors for substance dependence. The strongest impact on risk is associated with variation in the *CHRNA5* gene, where at least two mechanisms are at work: amino acid variation and altered mRNA expression levels. The risk allele of the non-synonymous variant (rs16969968; D398N) primarily occurs on the haplotype containing the low mRNA expression allele. In populations of European ancestry, there are approximately 50 highly correlated variants in the *CHRNA5-CHRNA3-CHRNB4* gene cluster and the adjacent *PSMA4* gene region that are associated with *CHRNA5* mRNA levels. It is not clear which of these variants contribute to the changes in *CHRNA5* transcript level. Because populations of African ancestry have reduced linkage disequilibrium among variants spanning this gene cluster, eQTL mapping in subjects of African ancestry could potentially aid in defining the functional variants that affect *CHRNA5* mRNA levels. We performed quantitative allele specific gene expression using frontal cortices derived from 49 subjects of African ancestry and 111 subjects of European ancestry. This method measures allele-specific transcript levels in the same individual, which eliminates other biological variation that occurs when comparing expression levels between different samples. This analysis confirmed that substance dependence associated variants have a direct *cis*-regulatory effect on *CHRNA5* transcript levels in human frontal cortices of African and European ancestry and identified 10 highly correlated variants, located in a 9 kb region, that are potential functional variants modifying *CHRNA5* mRNA expression levels.

## Introduction

Several genome-wide association studies have linked chromosome 15q24-q25.1, a region containing the genes encoding the α3, α5, and β4 subunits of neuronal nicotinic receptors, with nicotine dependence and smoking-related illnesses such as lung cancer, airflow obstruction, and chronic obstructive pulmonary disease [1]–[6]. In candidate gene association studies, variants in the *CHRNA5-A3-B4* gene cluster have been associated with nicotine dependence [7]–[14], smoking behaviors [15], [16], level of response to alcohol [17], age of initiation of drinking [15] and cocaine dependence [11], [18]. The strongest impact on the risk of substance abuse and lung cancer is associated with variation in *CHRNA5*.

Our previous studies showed there are at least two possible biological mechanisms accounting for these associations: an amino acid variation in exon 5 of *CHRNA5* (rs16969968; D398N), which likely alters protein structure and receptor function and variation in *CHRNA5* mRNA expression levels [8], [19], [20]. In European American populations, the nicotine dependence risk allele (minor allele) of the non-synonymous variant (rs16969968; D398N) primarily occurs on the haplotype containing the low mRNA expression allele of *CHRNA5*. The major allele at rs16969968 occurs on both high and low expression haplotypes. When the major allele occurs on the low mRNA expression haplotype of *CHRNA5*, the risk for nicotine dependence and lung cancer is significantly lower when compared to major alleles on the higher mRNA expression haplotype [19]. Lung cancer studies also demonstrated up-regulation of *CHRNA5* mRNA expression in lung adenocarcinomas, compared to normal lung tissue [21], [22]. In addition, *CHRNA5* mRNA expression in normal lung tissue was significantly associated with the genotype of rs16969968. mRNA expression level was about 2.5-fold lower in patients who are homozygous for the minor allele of rs16969968 than patients who are homozygous for the major allele [22].

There are approximately 50 variants spanning ∼83 kb within and flanking the *CHRNA5-A3-B4* gene cluster and the adjacent *PSMA4* gene that are highly correlated (D′≥0.9; r^2^≥0.7) with the variants (i.e. rs3841324, rs588765, rs880395) associated with *CHRNA5* mRNA levels in populations of European ancestry (Table S1). It is not clear which of these variants directly affect *CHRNA5* mRNA expression. Because populations of African ancestry have reduced linkage disequilibrium (LD) patterns across this gene cluster (http://hapmap.ncbi.nlm.nih.gov) (Table S1), the contrasting genetic architecture in Africans and Europeans can be leveraged to identify the functional variation most tightly linked to differences in *CHRNA5* mRNA expression. A previous study using quantitative allele specific gene expression in prefrontal cortex tissue from 59 Caucasians and 14 African Americans reported a cluster of 6 highly correlated SNPs located ∼13.5 kb upstream of the *CHRNA5* gene that accounted for the variability in mRNA expression [23]. Differential allelic expression of *CHRNA5* was also detected in normal lung tissue and in lung adenocarcinoma; two single nucleotide polymorphisms (rs55853698 and rs55781567) in the 5′UTR of *CHRNA5* were associated with significant imbalance in allelic expression ratio [24]. However, another study with 6 samples derived from human frontal cortex, amygdala or nucleus accumbens showed only one of the 6 samples revealed significant *CHRNA5* allelic expression imbalance in amygdala and nucleus accumbens, but not in frontal cortex [25]. The subject which showed allelic expression imbalance was heterozygous for 2 SNPs in ∼13.5 kb region upstream of the gene, but was homozygous for the variant (rs3841324) in the promoter region adjacent to *CHRNA5* transcription start site [25].

Our previous studies in frontal cortices of European ancestry demonstrated significant *cis*-regulatory effects on the *CHRNA5* mRNA levels. In contrast only modest variation in *CHRNA3* or *CHRNB4* mRNA levels were detected in our sample and these were not associated with SNP variation. In this study, we focused on *CHRNA5*. We first examined the *CHRNA5* mRNA variability in frontal cortices derived from 49 African Americans and confirmed our previous observation in other ethnic population. Further, we quantified *CHRNA5* allelic mRNA expression in frontal cortices derived from 66 European Australians, 45 European Americans, and 49 African Americans, to investigate the putative *cis*-regulatory variants that affect mRNA expression of this gene. Because several *cis*-regulatory variants for *CHRNA5* mRNA expression are located in the *PSMA4* gene region, we also examined the influence of these variants on *PSMA4* mRNA expression to clarify whether the *cis*-regulatory effect is specific for *CHRNA5* mRNA expression.

## Materials and Methods

### Study subjects

Three sets of postmortem brain tissues from the frontal cortex and one set of lymphoblastoid cells were tested in this study: (a) tissue derived from 49 unrelated African Americans was obtained from the National Institute of Child Health and Human Development (NICHD) brain and tissue bank for developmental disorders at the University of Maryland (http://medschool.umaryland.edu/btbank/); (b) tissue derived from 66 unrelated European Australians (35 alcoholics and 31 controls) was received from the Australian Brain Donor Programs NSW Tissue Resource Centre (http://sydney.edu.au/medicine/pathology/trc); (c) tissue derived from 45 unrelated, non-demented elderly European Americans was obtained from the Knight Alzheimer's Disease Research Center at Washington University (http://alzheimer.wustl.edu/); (d) cDNA and gDNA from 120 lymphoblastoid cell lines were obtained from the Rutgers University Cell and DNA Repository (http://www.rucdr.org/). These lymphoblastoid cell lines were generated from 60 unrelated African Americans and 60 unrelated European Americans, who were selected from the COGA dataset based on the genotypes of rs3841324 from our previous study [20]. In the African American subset, 24 subjects are homozygous for the major allele of rs3841324, 21 subjects are homozygous for the minor allele, and 15 subjects are heterozygous. In the European American subset, 21 subjects are homozygous for the major allele of rs3841324, 18 subjects are heterozygous, and 21 subjects are homozygous for the minor allele. The Washington University IRB reviewed the protocol using brain tissue and determined it was exempt from approval. The study using the COGA dataset was approved by IRB at all participating institutions. All data were analyzed anonymously.

### Genotyping

We used the Qiagen DNeasy Blood and Tissue Kit (Qiagen, Valencia, California, USA) to extract DNA from brain tissue. We used the PrimerPicker software (http://www.kbioscience.co.uk/primer-picker/) to design the assay and followed the protocol described in the KASPar SNP Genotyping System manual (http://www.kbioscience.co.uk) to run PCR reactions with an ABI GeneAmp PCR System 9700 (Applied Biosystems www.AppliedBiosystems.com). Genotypes were evaluated using an ABI instrument, 7900 HT Fast Real-Time PCR system. The 22-bp insertion/deletion variant, rs3841324 was genotyped using electrophoresis method described in our previous study [20]. We used Haploview software (v3.2) [26], to infer the LD structure of the genome in the region containing loci associated with *CHRNA5* expression in brain samples tested.

### Quantitative real-time total mRNA expression assay

We used the Qiagen RNeasy and Lipid Tissue kit (Qiagen, Valencia, CA, USA) to extract total RNA from brain tissue. RNA concentration was measured with a NanoDrop 2000 spectrophotometer (Thermo Scientific, Pittsburgh, Philadelphia, USA) and 5–10 ug total RNA was converted to cDNA with a High Capacity cDNA Archive kit (http://www.appliedbiosystems.com). TaqMan assays were used to quantify the total mRNA expression of *CHRNA5* (Hs00181248_m1; Applied Biosystems, CA, USA), and *PSMA4* (Hs01002583_m1) in human frontal cortices. Gene expression levels were assessed by real-time PCR using an ABI-7900HT Fast real-time PCR system. Each real-time PCR run included within-plate triplicates. Correction for sample-to-sample variation was done by simultaneously amplifying *GAPDH* (Hs02758991_g1) as a reference. We used the comparative Ct method to analyze total mRNA expression levels of *CHRNA5* and *PSMA4* and then normalized with *GAPDH* mRNA expression to obtain relative total mRNA expression level. A detailed protocol is described in Wang et al. [19], [20].

### Association analysis of genetic effect on CHRNA5 and PSMA4 total mRNA expression

To obtain a normal distribution, we log transformed relative total mRNA expression. Association between SNP genotype and relative total mRNA expression of *CHRNA5* and *PSMA4* was performed in PLINK [27] using linear regression under an additive genetic model. To minimize possible effects of sample heterogeneity, we performed our association analysis in subjects of each ancestral group separately. Similar to our observation in European Americans, postmortem interval (PMI  = 13.9±6.0 hour in samples of African ancestry; 22.5±14.7 hour in samples of European ancestry) had a weak effect on *CHRNA5* mRNA expression levels in brains of African ancestry and this variable was included as a covariate for association analysis. Age and gender did not affect *CHRNA5* expression and were not included as covariates. For *PSMA4* mRNA expression analysis, age had a weak effect in brains of European ancestry but not in samples of African ancestry; therefore this variable was included as a covariate for association analysis in the European ancestry datasets. PMI and gender did not influence *PSMA4* mRNA expression.

### Allele-specific expression (ASE)

Allelic mRNA expression level is determined by comparing the number of genomic DNA molecules for each allele with the number of allelic mRNA molecules within the same individual and same tissue, thus mRNA expression levels from the paternal and maternal alleles would be the same unless there are *cis*-regulatory variants that affect gene expression. We selected subjects with heterozygous genotypes for the coding SNP (rs16969968) and/or a SNP (rs615470) in the 3′UTR of *CHRNA5* for allele-specific expression measures. TaqMan genotyping assays for rs16969968 (Life technologies, C_26000428_20) and rs615470 (Life technologies, C_18757_10) are within the *CHRNA5* transcript, and were used for allele specific quantitative RT-PCR in each genomic DNA and cDNA. To enhance RT-PCR template, we pre-amplified gDNA and cDNA using primers flanking the region targeted by the TaqMan genotyping assay and ran a standard PCR reaction for 20 cycles. Primers used for rs16969968 pre-amplification are: forward primer 5′-CGCCTTTGGTCCGCAAGATA-3′ and reverse primer 5′-TGCTGATGGGGGAAGGTGGAG-3′. Primers used for rs615470 pre-amplification are: forward primer 5′- CAGATGATCCATTTGAACAGTTGGC-3′ and reverse primer 5′- TAGAGACGGGGTTTCTCCACGTTG-3′. Portions of pre-amplified cDNA from heterozygous samples for rs16969968 and/or rs615470 were arrayed in triplicate onto 384-well plates. As a control, a portion of pre-amplified gDNA from matched samples was also arrayed in duplicate onto the same 384-well plate. All reactions were evaluated on an ABI-7900HT Fast real-time PCR system under standard conditions.

### Determination of allelic mRNA expression imbalance (AEI)

The transcript level in each sample should be identical for allele 1 and allele 2 unless there is a *cis*-regulatory effect of one allele that changes its relative expression level. Therefore, one allele serves as the control for the other. To evaluate the amplification efficiency for allelic mRNA expression, we first set up a dilution series using one sample (cDNA or gDNA) that was homozygous for allele 1 and another sample that was homozygous for allele 2. The RT-PCR efficiency for each allele ranges from 95% to 100% with the cDNA sample. In the gDNA sample, the amplification efficiency ranges from 94% to 100%. Real-time data were analyzed using the comparative Ct method [28] and were corrected for the PCR efficiency of each assay. In each cDNA sample, the delta Ct (Ct value of allele 1 - Ct value of allele 2) value was calculated as an average of triplicate reactions. In gDNA samples, delta Ct values were calculated as an average of duplicate reactions. Only the samples with ≤10% standard errors in dCt were used for further analysis.

Allelic expression for each cDNA sample was normalized against the overall average ratio obtained for gDNA from each run. Subjects with greater than 2-fold difference in relative ASE (cDNA allelic expression/gDNA allelic expression) possess allelic expression imbalance (AEI). To verify our AEI determination threshold was accurate, we applied the Davies-Bouldin (DB) validity index [29]. The lowest values of the index reflect that within a bin the measurements are more similar to each other than the measurements in the other bins [29].

## Results

### Total CHRNA5 mRNA level in frontal cortex is strongly associated with variants located upstream of the gene in individuals of both African and European ancestry

**Figure 1:**
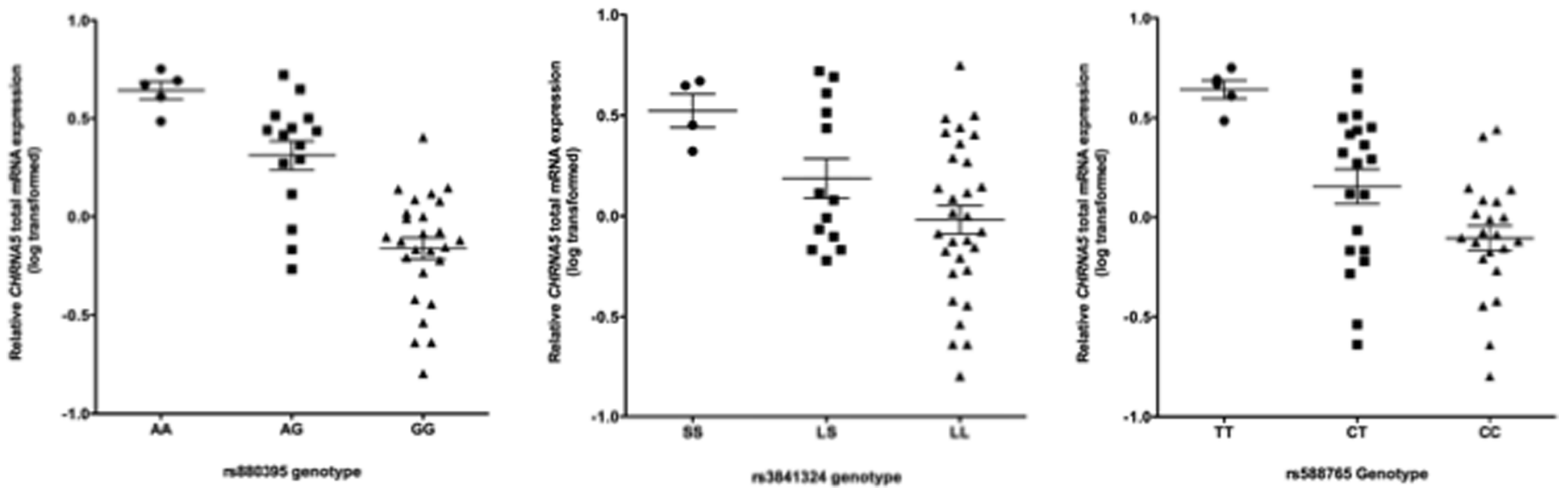
Minor allele of rs880395 (A allele), rs3841324 (S allele) and rs588765 (T allele) are associated with increased total mRNA expression of *CHRNA5* in human frontal cortex of African Americans.

Similar to our previous study in human brains of European ancestry [19], [20], the variability in *CHRNA5* total mRNA expression levels in frontal cortices of African ancestry is significantly associated with rs880395 (p = 7.27×10^−7^), rs3841324 (p = 6.94×10^−3^), and rs588765 (p = 1.10×10^−4^) (Table 1). Subjects homozygous for the minor allele at rs880395 and rs3841324, and rs588765 showed 5.4-fold, 2.6-fold, and 4.7-fold increase in *CHRNA5* mRNA expression levels compared to the major allele homozygotes, respectively (Figure 1).

**Table 1:** Association of *CHRNA5* total mRNA expression with variants within and flanking the *CHRNA5* gene.

SNP	chr 15 position (build 37)	Gene	Minor allele	Frontal cortex of European ancestry (PMI as a covariate)				Frontal cortex of African ancestry (PMI as a covariate)			
				N	BETA	STAT	P	N	BETA	STAT	P
rs12916483	78832397	−358 bp relative to AUG of *PSMA4*	A	93	0.66	8.3	9.92E-13	47	0.36	2.51	1.58E-02
rs4886571	78833758	intronic region of *PSMA4*	G	93	0.64	8.06	3.04E-12	47	0.6	4.72	2.40E-05
rs11858230	78835552	intronic region of *PSMA4*	A	93	0.64	8.01	3.88E-12	46	0.63	5	1.02E-05
rs8025429	78836362	intronic region of *PSMA4*	G	90	0.69	8.77	1.31E-13	45	0.63	4.99	1.12E-05
rs4887062	78837801	intronic region of *PSMA4*	G	94	0.64	8.18	1.60E-12	47	0.63	5.14	6.10E-06
rs8053	78841220	Exon 9 of *PSMA4*	T	93	0.64	8.01	3.87E-12	47	0.67	5.77	7.27E-07
rs1979907	78842239	−15.6 kb relative to AUG of *CHRNA5*	T	94	0.64	8.18	1.60E-12	47	0.67	5.77	7.27E-07
rs1979906	78842289	−15.6 kb relative to AUG of *CHRNA5*	C	94	0.64	8.18	1.60E-12	45	0.66	5.54	1.85E-06
rs1979905	78842374	−15.5 kb relative to AUG of *CHRNA5*	A	94	0.64	8.18	1.60E-12	47	0.67	5.77	7.27E-07
rs12907966	78843051	−14.8 kb relative to AUG of CHRNA5	T	93	0.66	8.42	5.57E-13	44	not polymorphic		
rs880395	78844356	−13.5 kb relative to AUG of *CHRNA5*	A	94	0.64	8.18	1.60E-12	47	0.67	5.77	7.27E-07
rs905740	78844386	−13.5 kb relative to AUG of *CHRNA5*	T	94	0.64	8.18	1.60E-12	47	0.67	5.77	7.27E-07
rs7164030	78844661	−13.2 kb relative to AUG of *CHRNA5*	G	92	0.64	8.06	3.21E-12	46	0.68	5.78	7.65E-07
rs4275821	78849541	−8.3 kb relative to AUG of CHRNA5	C	91	0.54	5.72	1.46E-07	47	0.61	5.21	4.84E-06
rs3841324	78857813	promoter region of *CHRNA5*	S	94	0.68	8.45	4.48E-13	47	0.39	2.83	6.94E-03
rs55853698	78857939	5′UTR of *CHRNA5*	G	92	−0.33	−3.34	1.22E-03	47	−0.12	−0.8	4.29E-01
rs588765	78865425	intronic region of *CHRNA5*	T	93	0.58	7.08	3.11E-10	47	0.54	4.25	1.10E-04
rs601079	78869579	intronic region of *CHRNA5*	T	92	0.58	7.1	2.89E-10	47	0.46	3.51	1.04E-03
rs16969968	78882925	Exon 5 of *CHRNA5*	A	94	−0.26	−2.64	9.83E-03	47	−0.11	−0.75	4.57E-01
rs615470	78885988	3′UTR of *CHRNA5*	T	94	0.4	4.36	3.40E-05	47	0.43	3.13	3.07E-03
rs578776	78888400	3′UTR of *CHRNA3*	A	92	−0.25	−2.04	4.40E-02	47	0.38	2.78	7.99E-03
rs3743078	78894759	intronic region of *CHRNA3*	C	94	−0.46	−4.03	1.16E-04	47	0.36	2.62	1.21E-02
rs6495308	78907656	intronic region of *CHRNA3*	C	93	−0.45	−3.92	1.73E-04	47	−0.35	−2.54	1.45E-02

*Bolded beta values indicate variants with significant association between minor alleles and increased mRNA expression.

The insertion-deletion variant rs3841324 within the putative promoter region of *CHRNA5* is highly correlated with rs880395 and rs588765 (r^2^>0.8; Figure S1a) in our brain samples of European ancestry, however it is very weakly correlated with rs880395 (r^2^ = 0.12) and rs588765 (r^2^ = 0.16) in brain samples of African ancestry (Figure S1b). rs588765 has a modest correlation with rs880395 in samples of African ancestry (r^2^ = 0.63; Figure S1b). To test whether the associations of *CHRNA5* mRNA expression with rs3841324 and with rs588765 are due to the LD with rs880395, we performed conditional analyses. When the effect of rs880395 on *CHRNA5* mRNA expression was included as a covariate, the association between rs3841324 or rs588765 and *CHRNA5* expression level was no longer significant in samples of African ancestry or in samples of European ancestry. Conversely, in conditional analysis controlling for the effect of rs3841324, the association of rs880395 with *CHRNA5* mRNA expression remains statistically significant in samples of African ancestry (p = 1.79×10^−5^) and in samples of European ancestry (p = 1.99×10^−3^). Similarly, conditional analysis showed rs880395 remains significantly associated with *CHRNA5* expression in both samples of African ancestry (p = 1.86×10^−5^) and European ancestry (1.38×10^−5^) after controlling for the effect of rs588765.

To further investigate eQTLs within and flanking the *CHRNA5* gene, we genotyped twenty additional SNPs including 2 variants (rs601079, rs615470) that are associated with *CHRNA5* total mRNA expression levels in brain tissues of European ancestry reported in our previous study [20], 5 tag SNPs (rs55853698, rs16969968, rs578776, rs3743078, rs6495308) from different LD bins in the *CHRNA5-A3-B4* gene cluster [19], and 12 SNPs highly correlated with rs880395 in the European population (r^2^ = 0.9). The linear regression results with postmortem interval as a covariate showed that the most significant variants associated with altered mRNA expression levels are located upstream of the *CHRNA5* transcript (Table 1).

### Tissue-specific effect on the variability of CHRNA5 mRNA levels

We tested whether eQTLs for *CHRNA5* expression found in human brain are also present in lymphoblastoid cells. We measured *CHRNA5* total mRNA expression levels in 60 lymphoblastoid cell lines from European Americans and 60 lymphoblastoid cell lines from African Americans and examined the effect of rs880395 and rs3841324 on expression. In cells from European Americans, both rs880395 (p = 1.02×10^−3^) and rs3841324 (p = 6.0×10^−4^) were significantly associated with variability of *CHRNA5* mRNA expression. However, the effect is much weaker and is in the opposite direction to the effect detected in frontal cortex. The minor alleles of rs880395 and rs3841324 are associated with increased mRNA expression of *CHRNA5* in human frontal cortex (Figure 1) but they are associated with decreased mRNA expression of *CHRNA5* in lymphoblastoid cells (Figure S2a). In cells from African Americans, no significant effect of rs880395 or rs3841324 on *CHRNA5* mRNA expression was observed (Figure S2b).

### Allelic expression imbalance (AEI) analysis in frontal cortices from European Americans/Australians

To further confirm the *cis*-regulatory effect on *CHRNA5* transcript levels and identify potential variants responsible for modulating mRNA expression, we performed allele specific expression (ASE) assays. We identified 49/111 and 55/111 brain samples derived from European Americans/Australians that are heterozygous for rs16969968 and rs615470, respectively.

**Figure 2:**
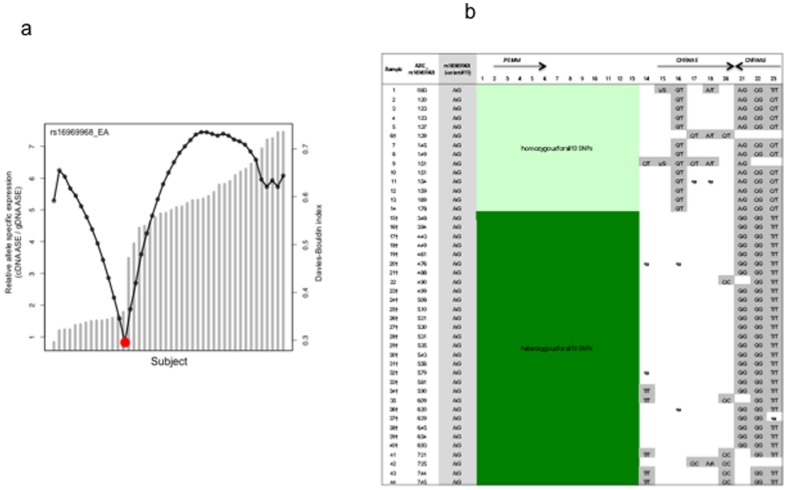
Allele specific expression (ASE) analysis with rs16969968 in 44 frontal cortices of European ancestry. 2a. Davies-Bouldin (DB) validity index analysis. Columns represent the ASE. Dotted line represents the DB validity index. The red dot indicates the optimal cutoff predicted by the DB index that coincides with ASE. 2b. Concordance of allelic expression variation with heterozygosity. Samples #1 to #14 which show relative allelic expression balance (*ASE<2) are homozygous for the 13 highlighted SNPs. Samples #15 to #44 which show relative allelic expression imbalance are heterozygous for all 13 SNPs. *See material and method for details; ASE: allele specific expression. † Indicates samples are also heterozygous for rs615470.

**Figure 3:**
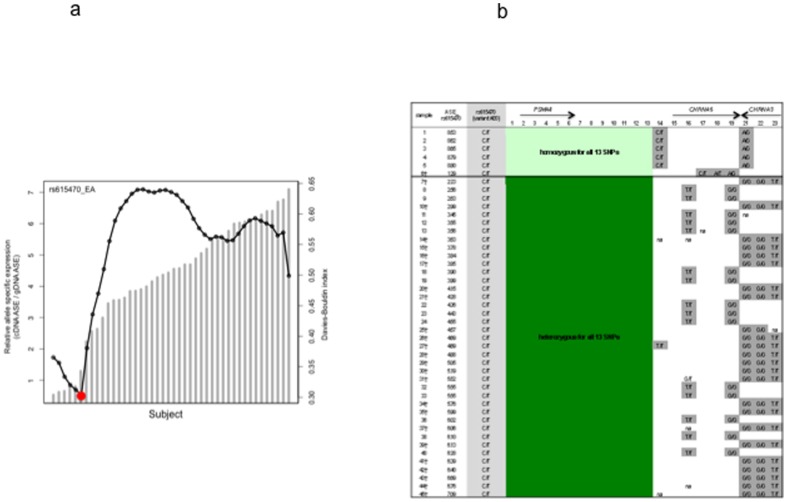
Allele specific expression (ASE) analysis with rs615470 in 45 frontal cortices of European ancestry. 3a. Davies-Bouldin (DB) validity index analysis. Columns represent the ASE. Dotted line represents the DB validity index. The red dot indicates the optimal cutoff predicted by the DB index that coincides with ASE. 3b. Concordance of allelic expression variation with heterozygosity. Samples #1 to #6 which show relative allelic expression balance (*ASE<2) are homozygous for the 13 highlighted SNPs. Samples #7 to #45 which showed relative allelic expression imbalance are heterozygous for all 13 SNPs. *See material and method for details; ASE: allele specific expression. † Indicates samples are also heterozygous for rs16969968.

We first applied the Davies-Bouldin (DB) validity index [29] to verify that relative ASE  = 2 is an appropriate threshold for AEI analysis. Our analysis showed the lowest DB index was at the threshold between ASE = 1.78 and ASE = 3.48 for rs16969968 (Figure 2a). The lowest DB index for rs615470 was at the threshold between ASE = 1.29 and ASE = 2.23 (Figure 3a). After excluding samples that failed in ASE assays and those with <90% genotyping success rate, 30 subjects showed more than 2-fold difference in relative ASE (AEI-positive) quantified with rs16969968; 14 subjects had ASE <2 and were determined to be AEI-negative (Figure 2b). Using ANOVA analysis, we detected strongly significant difference in relative ASE between AEI-positive and AEI-negative subsets (p-value = 1.85×10^−18^). With assay of rs615470, there were 39 AEI-positive subjects with 2.2-7.1-fold difference in relative ASE which is significantly different (p-value = 1.94×10^−9^) from the relative ASE in the AEI-negative subset (6 subjects) (Figure 3b).

The correlation of relative ASE measures between two experimental runs was 0.91 and 0.76 for rs16969968 and rs615470, respectively. We also compared the allelic mRNA expression in 26 samples that are heterozygous for both rs16969968 and rs615470 and observed consistent relative allelic expression (correlation  = 0.74) between the two markers (Figure S3).

### Allelic expression imbalance analysis in frontal cortices from African Americans

**Figure 4:**
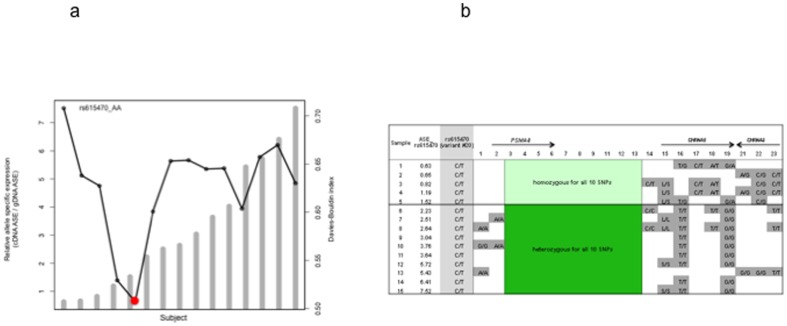
Allele specific expression (ASE) analysis with rs615470 in 15 frontal cortices of African ancestry. 4a. Davies-Bouldin (DB) validity index analysis. Columns represent the ASE. Dotted line represents the DB validity index. The red dot indicates the optimal d cutoff predicted by the DB index that coincides with ASE. 4b. Concordance of allelic expression variation with heterozygosity. Samples #1 to #5 which show relative allelic expression balance (*ASE<2) are homozygous for all 10 SNPs highlighted. Samples #6 to #15 which show relative allelic expression imbalance are heterozygous for all 10 SNPs. *See material and method for details; ASE: relative allele specific expression.

Because rs16969968 is rare in African Americans (minor allele frequency <0.05), we only used rs615470 to measure ASE. Applying the DB validity index, we detected the lowest DB index at the threshold between ASE = 1.52 and ASE = 2.53, which confirmed our threshold of relative ASE = 2 for AEI-positive and AEI-negative individuals (Figure 4a). Ten subjects were AEI-positive with 2.2 - 7.5-fold difference in relative ASE; 5 subjects were AEI-negative (Figure 4b). Our ANOVA analysis showed the difference of relative ASE between AEI-positive and AEI-negative subsets is statistically significant at p = 1.62×10^−3^. The correlation between two experiments of ASE assays is 0.97.

### Potential functional variants that affect CHRNA5 mRNA expression

**Figure 5:**
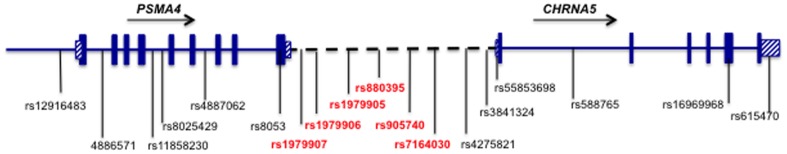
A schematic of *CHRNA5* and *PSMA4* gene structures. SNPs in red indicate potential functional eQTLs. Note: figure is not drawn to scale.

In this study we detected reproducible allelic expression of *CHRNA5* mRNA in frontal cortex of African Americans, Australians and European Americans. Therefore, we used allelic mRNA expression imbalance as a phenotypic trait to identify functional *cis*-acting variants. In frontal cortices of European ancestry, subjects who were AEI-positive (ASE >2) detected by either rs16969968 or rs615470 are heterozygous for 13 of the 23 variants genotyped (Figures 2b and 3b). These 13 variants, flanked by rs12916483 and rs4275821, are in a >12 kb region spanning from upstream of the *CHRNA5* transcription site (−8,521 bp relative to the AUG of *CHRNA5*) to upstream of the *PSMA4* transcription start site (−358 bp relative to the AUG of *PSMA4*) (Table 1, Figure 5). Subjects who were AEI-negative (ASE <2) are homozygous for all 13 variants. The concordance of AEI-positive with heterozygosity suggests that the difference in mRNA expression is affected by variants in this region.

We then tested the *cis*-acting effect in frontal cortex from individuals of African ancestry. All 10 subjects who were AEI-positive are heterozygous for 10 variants in a ∼9 kb region (−22.3 kb to −13.2 kb relative to the AUG of *CHRNA5*), defined by an intronic SNP (rs11858230) of *PSMA4* and rs7164030 located upstream of *CHRNA5* (Figures 4b and 5). Five subjects who were AEI-negative are homozygous for these 10 variants. This analysis suggests that the potential functional variants altering *CHRNA5* mRNA expression levels are likely located in this region.

### Association of SNP rs880395 and highly correlated variants with PSMA4 mRNA level in human frontal cortices

Because 4 of the 10 potentially functional variants associated with *CHRNA5* mRNA expression are within the *PSMA4* region, we tested whether these variants are also associated with *PSMA4* mRNA levels. We observed variability of *PSMA4* mRNA levels in both samples of African ancestry and European ancestry. However, the *PSMA4* mRNA expression observed in frontal cortices is not correlated with the level of *CHRNA5* mRNA expression (correlation  = −0.13, p = 0.2 in samples of European ancestry; correlation  = 0.03, p = 0.8 in samples of African ancestry). Using linear regression with age as a covariate, none of the 23 variants tested is associated with *PSMA4* mRNA levels in frontal cortices of European ancestry (Table S2). In brains of African ancestry, 4 of 22 polymorphic variants tested showed marginal association with *PSMA4* mRNA levels (5.96×10^−3^≤p≤3.91×10^−2^), but the association is no longer significant after correcting for multiple testing (q value ≥0.13).

## Discussion

The *cis*-regulatory effect on *CHRNA5* mRNA expression in frontal cortex from individuals of African ancestry is the same as observed in frontal cortex from individuals of European ancestry. In populations of European ancestry variants that alter *CHRNA5* mRNA expression levels are in an extensive linkage disequilibrium bin spanning ∼200 kb, with correlation coefficient (r^2^) of 0.7. In populations of African ancestry they fell into 7 bins (r^2^≥0.7) (Table S1), allowing us to map the functional variants influencing *CHRNA5* mRNA expression to a ∼9 kb region upstream of the *CHRNA5* transcription start site (−22.3 kb to −13.2 kb at AUG of *CHRNA5*).

Among 10 potential causal variants tested, 4 SNPs, including one synonymous SNP (rs8053), are within the *PSMA4* region. *PSMA4*, which encodes proteasome subunit alpha type-4, was reported as a strong candidate mediator of lung cancer cell growth [30]. Liu et al. [30] reported *PSMA4* mRNA levels were increased in lung tumors compared with normal lung tissues. However, no change in *PSMA4* mRNA expression was detected in another study of paired normal lung and lung adenocarcinoma tissue [21]. Our eQTL analysis showed none of the variants tested within and flanking *CHRNA5* and *PSMA4* is associated with *PSMA4* total mRNA levels (Table S2). In addition, we did not observe any correlation between *CHRNA5* and *PSMA4* mRNA expression in the brain samples tested. It is possible that the association between the 4 SNPs in *PSMA4* (rs11858230, rs8025429, rs4887062 and rs8053) and the changes in *CHRNA5* transcript levels results from the LD between these SNPs with rs880395.

The strong concordance between *CHRNA5* differential allele expression and heterozygosity detected in this study suggests that one or more of the 6 SNPs (rs1979907, rs1979906, rs1979905, rs880395, rs905740, and rs7164030), in a single LD bin (with r^2^≥0.94, D′ = 1 in populations of both African and European ancestry) spanning ∼2.5 kb at the 5′ distal region of the *CHRNA5* transcript are potential causal variants that alter the mRNA expression of this gene. This study reinforces previous findings that these 6 variants account for allelic expression differences in overall *CHRNA5* mRNA expression in human frontal cortices [23] and possibly in the human amygdala and nucleus accumbens [25].

Quantitative mRNA expression analysis in this study and previous studies have demonstrated that minor alleles of variants in the adjacent *CHRNA5* promoter region and/or 5′distal promoter region increase *CHRNA5* mRNA levels in post-mortem brain tissues and in lung tissue [19]–[21]. However, Doyle et al conducted a functional study that incorporated an 852 bp portion of the 5′ distal region of the *CHRNA5* promoter and showed strongly repressed transcription in cells with heterologous promoter constructs, compared to control vector [25]. No significant difference of the relative promoter activities was detected between the all-minor-allele haplotype construct and the all-major-allele haplotype construct [25]. The contradictory findings between the *in vitro* study and *ex vivo* study maybe explained by exclusion of the regions of the *CHRNA5* gene and adjacent promoter from the constructs used in Doyle et al study. Another possibility could be the tissue-specific nature of *CHRNA5* RNA expression. For example, we observed that the genetic effects on *CHRNA5* total mRNA expression in lymphoblastoid cells of European ancestry are in the opposite direction to that observed in human brains. Studies with promoter haplotypes, including rs3841324 and/or rs503464 within the *CHRNA5* promoter region and rs55853698 and rs55781567 within the 5′UTR, have shown that *CHRNA5* transcriptional activity was modulated by the promoter haplotype [21], [25]. It is possible that *CHRNA5* transcription is affected by variants in the 5′UTR, and adjacent and the 5′distal region of the *CHRNA5* promoter.

eQTLs are common in the human genome; some eQTLs are shared across tissues and some are tissue-specific [31]–[34]. A recent study integrating GWAS SNPs and gene expression profiles in blood and brain demonstrated that brain tissue is required for eQTL discovery for neurological diseases and psychiatric traits. For example, significant eQTLs that mapped to the Parkinson disease associated gene, *MAPT* were only detected in post-mortem brains but not in blood [32]. In this study, we detected a consistent *cis*-regulatory effect on the variability of *CHRNA5* mRNA levels in human frontal cortices of African and European ancestry but not in lymphoblastoid cell lines, which supports the importance of examining gene expression in brain, especially for psychiatric traits. Future studies examining other brain regions involved in the reward pathway may further define whether the eQTL for *CHRNA5* mRNA expression is limited to frontal cortex or is shared across brain regions.

A further limitation of this study is the absence of a direct assay of α5 protein in brain tissue. There are several caveats when mRNA transcript level is used as a proxy for functional receptor number. The mRNA level largely reflects the content of the somato-dendritic compartment, while it is very likely that most receptors incorporating the α5 subunit are located in the terminal regions of the cell [35]. Further, it is known that the number of receptors containing a subunit protein can change by up to 2-fold in the absence of a change in mRNA content (for example, a study by Pauly et al)[36]. Finally, the amount of α5 subunit protein available has been shown to strongly influence the assembly of receptors containing α4 and β2 subunits [37] and so the ultimate level of functional receptors containing the α5 subunit may be influenced by additional factors. Because of these factors it is not possible to directly link our measured effects on transcription to a predicted change in the availability of functional cell-surface receptors containing the α5 subunit. However, a reduction in transcript availability has the potential to have widespread effects on the number and subunit stoichiometry of surface receptors.

In summary, our allele specific expression analysis in human brains of African and European ancestry confirmed the *cis*-regulatory effect on *CHRNA5* mRNA expression and implicated variants in a 2.5 kb region upstream of *CHRNA5* transcription site as the putatively functional variants.

## Supporting Information

Figure S1 a. Linkage disequilibrium between variants in human brains of European ancestry. Number in each square represents the correlation (r^2^) between 2 variants genotyped in 111 frontal cortices included in this study. b. Linkage disequilibrium between variants in human brains of African ancestry. Number in each square represents the correlation (r^2^) between 2 variants genotyped in 49 frontal cortices included in this study.
(TIFF)

Figure S2 a. Minor allele of rs880395 (A allele) and rs3841324 (S allele) are associated with decreased relative *CHRNA5* total mRNA expression (*CHRNA5* total mRNA expression/*GAPDH* total mRNA expression) in lymphoblastoid cells of European Americans. Y-axis represents the log10 values of relative *CHRNA5* total mRNA expression. b. Minor allele of rs880395 (A allele) and rs3841324 (S allele) are not associated with relative *CHRNA5* total mRNA expression (*CHRNA5* total mRNA expression/*GAPDH* total mRNA expression) in lymphoblastoid cells of African Americans. Y-axis represents the log10 values of relative *CHRNA5* total mRNA expression.
(TIFF)

Figure S3 Correlation of relative allele specific expression (cDNA ASE/gDNA ASE) between rs16969968 and rs615470 in frontal cortex of European ancestry.
(TIFF)

Table S1 Linkage disequilibrium (represented by bin number) among SNPs associated with *CHRNA5* mRNA expression using HapMap release 2_22 data.
(DOCX)

Table S2 Association of relative *PSMA4* total mRNA expression (*PSMA4* total mRNA expression/*GAPDH* total mRNA expression) with variants within and flanking *CHRNA5* gene.
(DOCX)

## References

[1] Amos CI, Wu X, Broderick P, Gorlov IP, Gu J, et al (2008) Genome-wide association scan of tag SNPs identifies a susceptibility locus for lung cancer at 15q25.1. Nature genetics 40: 616–622. 18385676 10.1038/ng.109 PMC2713680

[2] Bierut LJ (2011) Genetic vulnerability and susceptibility to substance dependence. Neuron 69: 618–627. 21338875 10.1016/j.neuron.2011.02.015 PMC3095110

[3] Hung R, McKay J, Gaborieau V, Boffetta P, Hashibe M, et al (2008) A susceptibility locus for lung cancer maps to nicotinic acetylcholine receptor subunit genes on 15q25. Nature 452: 633–637. 18385738 10.1038/nature06885

[4] Thorgeirsson TE, Geller F, Sulem P, Rafnar T, Wiste A, et al (2008) A variant associated with nicotine dependence, lung cancer and peripheral arterial disease. Nature 452: 638–642. 18385739 10.1038/nature06846 PMC4539558

[5] Wang JC, Kapoor M, Goate AM (2012) The genetics of substance dependence. Annu Rev Genomics Hum Genet 13: 241–261. 22703173 10.1146/annurev-genom-090711-163844 PMC3474605

[6] Wilk JB, Shrine NR, Loehr LR, Zhao JH, Manichaikul A, et al (2012) Genome-wide association studies identify CHRNA5/3 and HTR4 in the development of airflow obstruction. Am J Respir Crit Care Med 186: 622–632. 22837378 10.1164/rccm.201202-0366OC PMC3480517

[7] Baker TB, Weiss RB, Bolt D, von Niederhausern A, Fiore MC, et al (2009) Human neuronal acetylcholine receptor A5-A3-B4 haplotypes are associated with multiple nicotine dependence phenotypes. Nicotine Tob Res 11: 785–796. 19436041 10.1093/ntr/ntp064 PMC2699926

[8] Bierut LJ, Stitzel JA, Wang JC, Hinrichs AL, Grucza RA, et al (2008) Variants in nicotinic receptors and risk for nicotine dependence. The American journal of psychiatry 165: 1163–1171. 18519524 10.1176/appi.ajp.2008.07111711 PMC2574742

[9] Saccone NL, Saccone SF, Hinrichs AL, Stitzel JA, Duan W, et al (2009) Multiple distinct risk loci for nicotine dependence identified by dense coverage of the complete family of nicotinic receptor subunit (CHRN) genes. American journal of medical genetics Part B, Neuropsychiatric genetics : the official publication of the International Society of Psychiatric Genetics 150B: 453–466. 10.1002/ajmg.b.30828 PMC2693307 19259974

[10] Saccone SF, Hinrichs AL, Saccone NL, Chase GA, Konvicka K, et al (2007) Cholinergic nicotinic receptor genes implicated in a nicotine dependence association study targeting 348 candidate genes with 3713 SNPs. Human molecular genetics 16: 36–49. 17135278 10.1093/hmg/ddl438 PMC2270437

[11] Sherva R, Kranzler HR, Yu Y, Logue MW, Poling J, et al (2010) Variation in nicotinic acetylcholine receptor genes is associated with multiple substance dependence phenotypes. Neuropsychopharmacology 35: 1921–1931. 20485328 10.1038/npp.2010.64 PMC3055642

[12] Spitz MR AC, Dong Q, Lin J, Wu X (2008) The CHRNA5-A3 region on chromosome 15q24-25.1 is a risk factor both for nicotine dependence and for lung cancer. J Natl Cancer Inst 100: 1552–1556. 18957677 10.1093/jnci/djn363 PMC2720751

[13] Stevens VL, Bierut LJ, Talbot JT, Wang JC, Sun J, et al (2008) Nicotinic receptor gene variants influence susceptibility to heavy smoking. Cancer epidemiology, biomarkers & prevention : a publication of the American Association for Cancer Research, cosponsored by the American Society of Preventive Oncology 17: 3517–3525. 10.1158/1055-9965.EPI-08-0585 PMC2614129 19029397

[14] Weiss RB, Baker TB, Cannon DS, von Niederhausern A, Dunn DM, et al (2008) A candidate gene approach identifies the CHRNA5-A3-B4 region as a risk factor for age-dependent nicotine addiction. PLoS genetics 4: e1000125. 18618000 10.1371/journal.pgen.1000125 PMC2442220

[15] Schlaepfer I, Hoft NR, Collins AC, Corley RP, Hewitt JK, et al (2008) The CHRNA5/A3/B4 gene cluster variability as an important determinant of early alcohol and tobacco initiation in young adults. Biol Psychiatry 63: 1039–1046. 18163978 10.1016/j.biopsych.2007.10.024 PMC2526976

[16] Sherva R, Wilhelmsen K, Pomerleau CS, Chasse SA, Rice JP, et al (2008) Association of a single nucleotide polymorphism in neuronal acetylcholine receptor subunit alpha 5 (CHRNA5) with smoking status and with ‘pleasurable buzz’ during early experimentation with smoking. Addiction 103: 1544–1552. 18783506 10.1111/j.1360-0443.2008.02279.x PMC2582398

[17] Joslyn G, Brush G, Robertson M, Smith TL, Kalmijn J, et al (2008) Chromosome 15q25.1 genetic markers associated with level of response to alcohol in humans. Proc Natl Acad Sci U S A 105: 20368–20373. 19064933 10.1073/pnas.0810970105 PMC2629302

[18] Grucza RA, Wang JC, Stitzel JA, Hinrichs AL, Saccone SF, et al (2008) A risk allele for nicotine dependence in CHRNA5 is a protective allele for cocaine dependence. Biol Psychiatry 64: 922–929. 18519132 10.1016/j.biopsych.2008.04.018 PMC2582594

[19] Wang JC, Cruchaga C, Saccone NL, Bertelsen S, Liu P, et al (2009) Risk for nicotine dependence and lung cancer is conferred by mRNA expression levels and amino acid change in CHRNA5. Human molecular genetics 18: 3125–3135. 19443489 10.1093/hmg/ddp231 PMC2714722

[20] Wang JC, Grucza R, Cruchaga C, Hinrichs AL, Bertelsen S, et al (2009) Genetic variation in the CHRNA5 gene affects mRNA levels and is associated with risk for alcohol dependence. Molecular psychiatry 14: 501–510. 18414406 10.1038/mp.2008.42 PMC4381434

[21] Falvella FS, Galvan A, Colombo F, Frullanti E, Pastorino U, et al (2010) Promoter polymorphisms and transcript levels of nicotinic receptor CHRNA5. Journal of the National Cancer Institute 102: 1366–1370. 20733116 10.1093/jnci/djq264

[22] Falvella FS, Galvan A, Frullanti E, Spinola M, Calabro E, et al (2009) Transcription deregulation at the 15q25 locus in association with lung adenocarcinoma risk. Clin Cancer Res 15: 1837–1842. 19223495 10.1158/1078-0432.CCR-08-2107

[23] Smith RM, Alachkar H, Papp AC, Wang D, Mash DC, et al (2011) Nicotinic alpha5 receptor subunit mRNA expression is associated with distant 5′ upstream polymorphisms. Eur J Hum Genet 19: 76–83. 20700147 10.1038/ejhg.2010.120 PMC2995013

[24] Falvella FS, Alberio T, Noci S, Santambrogio L, Nosotti M, et al (2013) Multiple isoforms and differential allelic expression of CHRNA5 in lung tissue and lung adenocarcinoma. Carcinogenesis 34: 1281–1285. 23430818 10.1093/carcin/bgt062

[25] Doyle GA, Wang MJ, Chou AD, Oleynick JU, Arnold SE, et al (2011) In vitro and ex vivo analysis of CHRNA3 and CHRNA5 haplotype expression. PLoS One 6: e23373. 21858091 10.1371/journal.pone.0023373 PMC3155531

[26] Barrett JC, Fry B, Maller J, Daly MJ (2005) Haploview: analysis and visualization of LD and haplotype maps. Bioinformatics 21: 263–265. 15297300 10.1093/bioinformatics/bth457

[27] Purcell SNB, Todd-Brown K, Thomas L, Ferreira MA, Bender D, et al (2007) PLINK: a tool set for whole-genome association and population-based linkage analyses. Am J Hum Genet 81: 559–575. 17701901 10.1086/519795 PMC1950838

[28] Muller PY, Janovjak H, Miserez AR, Dobbie Z (2002) Processing of gene expression data generated by quantitative real-time RT-PCR. Biotechniques 32: : 1372–1374, 1376, 1378–1379. 12074169

[29] Davies DL, Bouldin DW (1979) A cluster separation measure. IEEE Trans Pattern Anal Mach Intell 1: 224–227. 21868852

[30] Liu Y, Liu P, Wen W, James MA, Wang Y, et al (2009) Haplotype and cell proliferation analyses of candidate lung cancer susceptibility genes on chromosome 15q24-25.1. Cancer Res 69: 7844–7850. 19789337 10.1158/0008-5472.CAN-09-1833 PMC2846106

[31] Brown CD, Mangravite LM, Engelhardt BE (2013) Integrative Modeling of eQTLs and Cis-Regulatory Elements Suggests Mechanisms Underlying Cell Type Specificity of eQTLs. PLoS genetics 9: e1003649. 23935528 10.1371/journal.pgen.1003649 PMC3731231

[32] Hernandez DG, Nalls MA, Moore M, Chong S, Dillman A, et al (2012) Integration of GWAS SNPs and tissue specific expression profiling reveal discrete eQTLs for human traits in blood and brain. Neurobiol Dis 47: 20–28. 22433082 10.1016/j.nbd.2012.03.020 PMC3358430

[33] Miao X, Leon TY, Ngan ES, So MT, Yuan ZW, et al (2010) Reduced RET expression in gut tissue of individuals carrying risk alleles of Hirschsprung's disease. Human molecular genetics 19: 1461–1467. 20089534 10.1093/hmg/ddq020

[34] Nica AC, Parts L, Glass D, Nisbet J, Barrett A, et al (2011) The architecture of gene regulatory variation across multiple human tissues: the MuTHER study. PLoS genetics 7: e1002003. 21304890 10.1371/journal.pgen.1002003 PMC3033383

[35] Dani JA, Bertrand D (2007) Nicotinic acetylcholine receptors and nicotinic cholinergic mechanisms of the central nervous system. Annu Rev Pharmacol Toxicol 47: 699–729. 17009926 10.1146/annurev.pharmtox.47.120505.105214

[36] Pauly JR, Marks MJ, Robinson SF, van de Kamp JL, Collins AC (1996) Chronic nicotine and mecamylamine treatment increase brain nicotinic receptor binding without changing alpha 4 or beta 2 mRNA levels. J Pharmacol Exp Ther 278: 361–369. 8764371

[37] Chatterjee S, Santos N, Holgate J, Haass-Koffler CL, Hopf FW, et al (2013) The alpha5 subunit regulates the expression and function of alpha4*-containing neuronal nicotinic acetylcholine receptors in the ventral-tegmental area. PLoS One 8: e68300. 23869214 10.1371/journal.pone.0068300 PMC3712017

