# Supplementary figures and images for: Cis-Regulatory Variants Affect CHRNA5 mRNA Expression in Populations of African and European Ancestry
Source: PLoS One. 2013 Nov 26;8(11):e80204. doi: 10.1371/journal.pone.0080204 (PMC3841173; doi:10.1371/journal.pone.0080204)

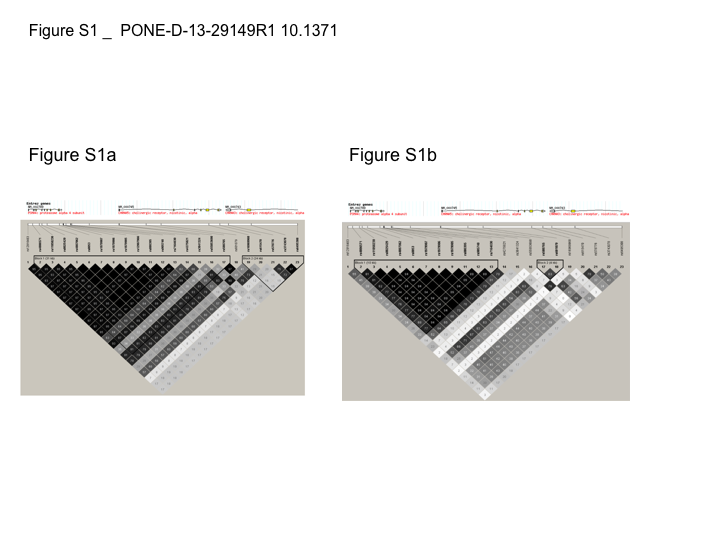

Supplement: Figure S1 — a. Linkage disequilibrium between variants in human brains of European ancestry. Number in each square represents the correlation (r2) between 2 variants genotyped in 111 frontal cortices included in this study. b. Linkage disequilibrium between variants in human brains of African ancestry. Number in each square represents the correlation (r2) between 2 variants genotyped in 49 frontal cortices included in this study. (TIFF) [file pone.0080204.s001.tif]

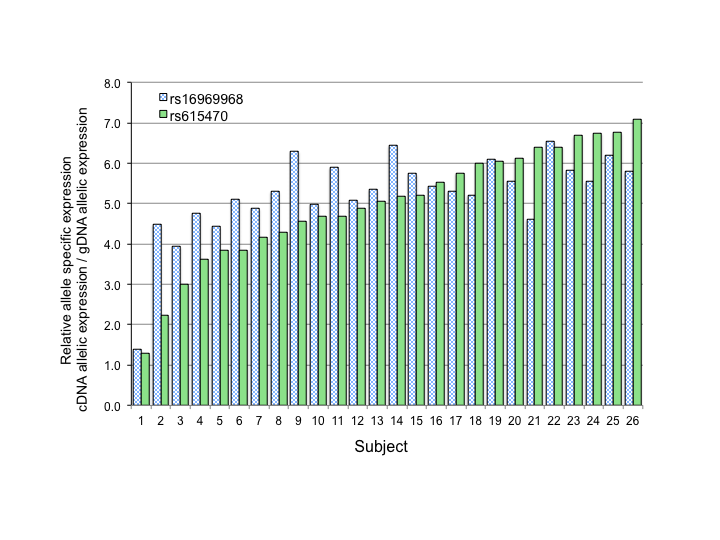

Supplement: Figure S3 — Correlation of relative allele specific expression (cDNA ASE/gDNA ASE) between rs16969968 and rs615470 in frontal cortex of European ancestry. (TIFF) [file pone.0080204.s003.tif]
