# Supplementary material for: Cis-Regulatory Variants Affect CHRNA5 mRNA Expression in Populations of African and European Ancestry
Source: PLoS One. 2013 Nov 26;8(11):e80204. doi: 10.1371/journal.pone.0080204 (PMC3841173; doi:10.1371/journal.pone.0080204)
Supplement: Table S1 — Linkage disequilibrium (represented by bin number) among SNPs associated with CHRNA5 mRNA expression using HapMap release 2_22 data. (DOCX) [file pone.0080204.s004.docx]

**Table S1. Linkage disequilibrium (represented by bin number) among SNPs associated with *CHRNA5* mRNA expression using HapMap release 2_22 data.**

| SNP id | Position (hg19) | CEU_ MAF | CEU_ r2=0.7 | CEU_ r2=0.8 | CEU_ r2=0.9 | CEU_ r2=1.0 | YRI_ MAF | YRI_ r2=0.7 | YRI_ r2=0.8 | YRI_ r2=0.9 | YRI_ r2=1.0 |
| --- | --- | --- | --- | --- | --- | --- | --- | --- | --- | --- | --- |
| rs12591557 | 78811732 | 0.36 | 1 | 1 | 1 | 1 | 0.12 | 1 | 1 | 1 | 1 |
| rs12914694 | 78814444 | 0.36 | 1 | 1 | 1 | 1 | 0.05 | 2 | 2 | 2 | 2 |
| rs1504545 | 78818471 | 0.36 | 1 | 1 | 1 | 1 | 0.10 | 1 | 1 | 3 | 3 |
| rs952215 | 78819153 | 0.36 | 1 | 1 | 1 | 1 | 0.10 | 1 | 1 | 3 | 3 |
| rs952216 | 78819202 | 0.36 | 1 | 1 | 1 | 1 | 0.09 | 1 | 1 | 3 | 4 |
| rs12902493 | 78819275 | 0.36 | 1 | 1 | 1 | 1 | 0.10 | 1 | 1 | 3 | 3 |
| rs11636131 | 78821606 | 0.36 | 1 | 1 | 1 | 1 | 0.11 | 1 | 1 | 4 | 5 |
| rs11632604 | 78821914 | 0.36 | 1 | 1 | 1 | 1 | 0.11 | 1 | 1 | 4 | 5 |
| rs12910289 | 78822065 | 0.36 | 1 | 1 | 1 | 1 | 0.11 | 1 | 1 | 4 | 5 |
| rs1504546 | 78824235 | 0.36 | 1 | 1 | 1 | 1 | 0.11 | 1 | 1 | 4 | 5 |
| rs12906951 | 78825562 | 0.36 | 1 | 1 | 1 | 1 | 0.11 | 1 | 1 | 4 | 5 |
| rs12916999 | 78826912 | 0.36 | 1 | 1 | 1 | 1 | 0.10 | 1 | 1 | 4 | 6 |
| rs12915366 | 78831753 | 0.34 | 1 | 1 | 1 | 2 | 0.11 | 1 | 1 | 4 | 5 |
| rs12916483 | 78832397 | 0.36 | 1 | 1 | 1 | 1 | 0.11 | 1 | 1 | 4 | 5 |
| rs3813572 | 78832588 | 0.36 | 1 | 1 | 1 | 1 | 0.11 | 1 | 1 | 4 | 5 |
| rs3813571 | 78832792 | 0.36 | 1 | 1 | 1 | 1 | 0.11 | 1 | 1 | 4 | 5 |
| rs4886571 | 78833758 | 0.36 | 1 | 1 | 1 | 1 | 0.23 | 3 | 3 | 5 | 7 |
| rs4243083 | 78833830 | 0.36 | 1 | 1 | 1 | 1 | 0.23 | 3 | 3 | 5 | 7 |
| rs2292117 | 78834689 | 0.36 | 1 | 1 | 1 | 1 | 0.17 | 4 | 4 | 6 | 8 |
| rs11858230 | 78835552 | 0.35 | 1 | 1 | 1 | 3 | 0.16 | 4 | 4 | 6 | 9 |
| rs8025429 | 78836362 | 0.36 | 1 | 1 | 1 | 1 | 0.17 | 4 | 4 | 6 | 8 |
| rs4887062 | 78837801 | 0.37 | 1 | 1 | 1 | 4 | 0.17 | 4 | 4 | 6 | 8 |
| rs4887063 | 78839715 | 0.36 | 1 | 1 | 1 | 1 | 0.18 | 4 | 4 | 6 | 10 |
| rs8053 | 78841220 | 0.36 | 1 | 1 | 1 | 1 | 0.18 | 4 | 4 | 6 | 10 |
| rs1979907 | 78842239 | 0.37 | 1 | 1 | 1 | 5 | 0.18 | 4 | 4 | 6 | 10 |
| rs1979906 | 78842289 | 0.37 | 1 | 1 | 1 | 5 | 0.18 | 4 | 4 | 6 | 10 |
| rs1979905 | 78842374 | 0.37 | 1 | 1 | 1 | 5 | 0.18 | 4 | 4 | 6 | 10 |
| rs12907966 | 78843051 | 0.37 | 1 | 1 | 1 | 5 | 0.05 | 2 | 2 | 2 | 2 |
| rs880395 | 78844356 | 0.37 | 1 | 1 | 1 | 5 | 0.18 | 4 | 4 | 6 | 10 |
| rs7164030 | 78844661 | 0.37 | 1 | 1 | 1 | 5 | 0.18 | 4 | 4 | 6 | 10 |
| rs4275821 | 78849541 | 0.33 | 1 | 1 | 2 | 6 | 0.18 | 4 | 4 | 7 | 11 |
| rs7173512 | 78849914 | 0.33 | 1 | 1 | 2 | 6 | 0.18 | 4 | 4 | 7 | 11 |
| rs588765 | 78865425 | 0.38 | 1 | 2 | 3 | 7 | 0.22 | 5 | 5 | 8 | 12 |
| rs6495306 | 78865893 | 0.38 | 1 | 2 | 3 | 7 | 0.23 | 5 | 5 | 8 | 13 |
| rs601079 | 78869579 | 0.38 | 1 | 2 | 3 | 7 | 0.43 | 6 | 6 | 9 | 14 |
| rs495956 | 78869930 | 0.33 | 1 | 2 | 4 | 8 | 0.43 | 6 | 6 | 9 | 14 |
| rs680244 | 78871288 | 0.38 | 1 | 2 | 3 | 7 | 0.43 | 6 | 6 | 9 | 14 |
| rs621849 | 78872861 | 0.38 | 1 | 2 | 3 | 7 | 0.43 | 6 | 6 | 9 | 14 |
| rs692780 | 78876505 | 0.33 | 1 | 2 | 4 | 8 | 0.20 | 5 | 5 | 10 | 15 |
| rs11637635 | 78877150 | 0.33 | 1 | 2 | 4 | 8 | 0.23 | 5 | 5 | 8 | 16 |
| rs481134 | 78877563 | 0.38 | 1 | 2 | 3 | 7 | 0.23 | 5 | 5 | 8 | 16 |
| rs555018 | 78879242 | 0.38 | 1 | 2 | 3 | 7 | 0.23 | 5 | 5 | 8 | 17 |
| rs17408276 | 78881618 | 0.33 | 1 | 2 | 4 | 9 | 0.05 | 7 | 7 | 11 | 18 |
| rs514743 | 78884227 | 0.33 | 1 | 2 | 4 | 9 | 0.20 | 5 | 5 | 10 | 15 |
| rs615470 | 78885988 | 0.33 | 1 | 2 | 4 | 9 | 0.34 | 8 | 8 | 12 | 19 |
| rs660652 | 78887832 | 0.33 | 1 | 2 | 4 | 9 | 0.23 | 5 | 5 | 8 | 20 |
| rs6495307 | 78890321 | 0.37 | 1 | 2 | 3 | 10 | 0.34 | 8 | 8 | 12 | 19 |
| rs3743077 | 78894896 | 0.37 | 1 | 3 | 5 | 11 | 0.07 | 9 | 9 | 13 | 21 |

MAF: minor allele frequency; CEU: Caucasian population; YRI: Yoruba population
