# Supplementary material for: Cis-Regulatory Variants Affect CHRNA5 mRNA Expression in Populations of African and European Ancestry
Source: PLoS One. 2013 Nov 26;8(11):e80204. doi: 10.1371/journal.pone.0080204 (PMC3841173; doi:10.1371/journal.pone.0080204)
Supplement: Table S2 — Association of relative PSMA4 total mRNA expression (PSMA4 total mRNA expression/GAPDH total mRNA expression) with variants within and flanking CHRNA5 gene. (DOCX) [file pone.0080204.s005.docx]

**Table S2. Association of relative *PSMA4* total mRNA expression (PSMA4 total mRNA expression / *GAPDH* total mRNA expression) with variants within and flanking *CHRNA5* gene.**

| **SNP#** | **SNP** | **chr 15 position (build 37)** | **Gene** | **Frontal cortex of European ancestry (age as a covariate)** | | | **Frontal cortex of African ancestry** | | |
| --- | --- | --- | --- | --- | --- | --- | --- | --- | --- |
|  |  |  |  | **N** | **BETA** | **P** | **N** | **BETA** | **P** |
| 1 | rs12916483 | 78832397 | −358 bp relative to AUG of *PSMA4* | 96 | -0.19 | 7.07E-02 | 48 | -0.06 | 1.22E-01 |
| 2 | rs4886571 | 78833758 | intronic region of *PSMA4* | 96 | -0.16 | 1.22E-01 | 48 | -0.07 | 5.44E-02 |
| 3 | rs11858230 | 78835552 | intronic region of *PSMA4* | 99 | -0.16 | 1.10E-01 | 47 | -0.08 | 5.03E-02 |
| 4 | rs8025429 | 78836362 | intronic region of *PSMA4* | 97 | -0.16 | 1.14E-01 | 47 | -0.08 | 3.12E-02 |
| 5 | rs4887062 | 78837801 | intronic region of *PSMA4* | 99 | -0.16 | 1.10E-01 | 49 | -0.07 | 3.91E-02 |
| 6 | rs8053 | 78841220 | Exon 9 of *PSMA4* | 95 | -0.19 | 7.19E-02 | 48 | -0.07 | 6.26E-02 |
| 7 | rs1979907 | 78842239 | −15.6 kb relative to AUG of *CHRNA5* | 99 | -0.16 | 1.10E-01 | 48 | -0.07 | 6.26E-02 |
| 8 | rs1979906 | 78842289 | −15.6 kb relative to AUG of *CHRNA5* | 99 | -0.16 | 1.10E-01 | 47 | -0.05 | 2.19E-01 |
| 9 | rs1979905 | 78842374 | −15.5 kb relative to AUG of *CHRNA5* | 99 | -0.16 | 1.10E-01 | 49 | -0.07 | 6.46E-02 |
| 10 | rs12907966 | 78843051 | −14.8 kb relative to AUG of CHRNA5 | 96 | -0.17 | 9.81E-02 | 46 | not polymorphic | |
| 11 | rs880395 | 78844356 | −13.5 kb relative to AUG of *CHRNA5* | 99 | -0.16 | 1.10E-01 | 48 | -0.07 | 6.26E-02 |
| 12 | rs905740 | 78844386 | −13.5 kb relative to AUG of *CHRNA5* | 98 | -0.17 | 9.32E-02 | 49 | -0.07 | 7.73E-02 |
| 13 | rs7164030 | 78844661 | −13.2 kb relative to AUG of *CHRNA5* | 97 | -0.15 | 1.43E-01 | 47 | -0.06 | 9.49E-02 |
| 14 | rs4275821 | 78849541 | −8.3 kb relative to AUG of CHRNA5 | 94 | -0.19 | 6.48E-02 | 49 | -0.05 | 1.14E-01 |
| 15 | rs3841324 | 78857813 | promoter region of *CHRNA5* | 99 | -0.18 | 8.22E-02 | 49 | -0.01 | 7.76E-01 |
| 16 | rs55853698 | 78857939 | 5'UTR of *CHRNA5* | 94 | 0.06 | 5.55E-01 | 49 | 0.10 | 1.20E-01 |
| 17 | rs588765 | 78865425 | intronic region of *CHRNA5* | 98 | -0.15 | 1.59E-01 | 49 | -0.08 | 3.32E-02 |
| 18 | rs601079 | 78869579 | intronic region of *CHRNA5* | 97 | -0.14 | 1.72E-01 | 49 | -0.09 | 5.96E-03 |
| 19 | rs16969968 | 78882925 | Exon 5 of *CHRNA5* | 99 | 0.07 | 5.15E-01 | 49 | 0.10 | 1.35E-01 |
| 20 | rs615470 | 78885988 | 3'UTR of *CHRNA5* | 99 | -0.09 | 3.99E-01 | 49 | -0.05 | 9.64E-02 |
| 21 | rs578776 | 78888400 | 3'UTR of *CHRNA3* | 97 | 0.06 | 5.56E-01 | 48 | -0.03 | 3.54E-01 |
| 22 | rs3743078 | 78894759 | intronic region of *CHRNA3* | 99 | 0.10 | 3.42E-01 | 49 | -0.04 | 2.77E-01 |
| 23 | rs6495308 | 78907656 | intronic region of *CHRNA3* | 97 | 0.10 | 3.23E-01 | 48 | 0.08 | 6.41E-02 |
